# The Benefit of Attention-to-Memory Depends on the Interplay of Memory Capacity and Memory Load

**DOI:** 10.3389/fpsyg.2018.00184

**Published:** 2018-02-19

**Authors:** Sung-Joo Lim, Malte Wöstmann, Frederik Geweke, Jonas Obleser

**Affiliations:** ^1^Department of Psychology, University of Lübeck, Lübeck, Germany; ^2^Max Planck Institute for Human Cognitive and Brain Sciences, Leipzig, Germany

**Keywords:** auditory working memory, retrospective attention, perceptual precision, psychophysical modeling, individual differences

## Abstract

Humans can be cued to attend to an item in memory, which facilitates and enhances the perceptual precision in recalling this item. Here, we demonstrate that this facilitating effect of attention-to-memory hinges on the overall degree of memory load. The benefit an individual draws from attention-to-memory depends on her overall working memory performance, measured as sensitivity (*d′*) in a retroactive cue (retro-cue) pitch discrimination task. While listeners maintained 2, 4, or 6 auditory syllables in memory, we provided valid or neutral retro-cues to direct listeners’ attention to one, to-be-probed syllable in memory. Participants’ overall memory performance (i.e., perceptual sensitivity *d′*) was relatively unaffected by the presence of valid retro-cues across memory loads. However, a more fine-grained analysis using psychophysical modeling shows that valid retro-cues elicited faster pitch-change judgments and improved perceptual precision. Importantly, as memory load increased, listeners’ overall working memory performance correlated with inter-individual differences in the degree to which precision improved (*r* = 0.39, *p* = 0.029). Under high load, individuals with low working memory profited least from attention-to-memory. Our results demonstrate that retrospective attention enhances perceptual precision of attended items in memory but listeners’ optimal use of informative cues depends on their overall memory abilities.

## Introduction

Internal representations of perceptual information held in memory are not perfectly precise, but inherently noisy – that is, our neural representations do not perfectly match the sensory information (Green and Swets, 1966; Wilken and Ma, 2004; Bays and Husain, 2008; Ma et al., 2014; Bays, 2015). Selective attention to the relevant representation in memory can effectively reduce such noise, by neurally prioritizing the attended representation (Serences and Kastner, 2014; see below for discussion of the possible mechanisms). This attentional enhancement of relevant memory items is referred to as *retrospective attention* (Griffin and Nobre, 2003; Gazzaley and Nobre, 2012).

Particularly, auditory memory representations pose an important case for selective attention: acoustic signals are often embedded in a noisy sound mixture, leading to imprecise representation in auditory memory. Thus, auditory memory should benefit from selective attention to relevant signals in midst of irrelevant noise (Gazzaley and Nobre, 2012; Wilsch and Obleser, 2016). Accordingly, retrospective attention facilitates the recall of attended versus unattended memory items (e.g., Griffin and Nobre, 2003; Pertzov et al., 2013; Lim et al., 2015).

Generally, selective attention strongly interacts with working memory (e.g., Craik and Levy, 1976; Awh and Jonides, 2001; Awh et al., 2006): overlapping neural resources of top–down control may operate both processes (see Gazzaley and Nobre, 2012 for a review), and individuals’ attentional control abilities predict working memory capacity (Fukuda and Vogel, 2009; Unsworth et al., 2014; Gaspar et al., 2016). Likewise, rather than a capacity-limited storage (Luck and Vogel, 1997; Cowan, 2001), working memory is increasingly considered as a flexible cognitive resource that can be allocated or distributed across items held in memory (Ma et al., 2014; Bays, 2015). Selective attention biases this resource allocation (Awh and Jonides, 2001; Gazzaley and Nobre, 2012), and the amount of allocated resource determines the precision of items in memory (Bays and Husain, 2008; van den Berg et al., 2012). This view suggests that retrospective attention requires reallocation/redistribution of limited cognitive resources to attended memory items.

Among multiple underlying mechanisms of retrospective attentional benefits found in visual studies (see Souza and Oberauer, 2016 for a review), the current work specifically focuses on one potential mechanism: retrospective attention enhances representational precision of the attended compared to unattended items in memory (Lepsien and Nobre, 2007; Lewis-Peacock et al., 2012; Rerko and Oberauer, 2013). A recent study in the auditory domain supports this account that retrospective attention enhances representational precision of auditory items in memory (Lim et al., 2015). Using a psychophysical modeling approach, this study demonstrated that with valid (vs. neutral) retro-cues, participants judged the pitch of cued syllables with greater precision. Neurally, valid versus neutral cues elicited two distinct electrophysiological signatures (i.e., enhancement of sustained negativity and alpha oscillatory power), both associated with increased demands on attention allocation and cognitive/memory load. Thus, rather than valid retro-cue reduces cognitive load by removing unattended items from memory, the use of the potentially beneficial valid retro-cue requires cognitive resources; that is, there is a neural cost for re-allocating attention to the cued item in memory, which enhances its representation. However, this evidence was gathered only under low memory load of two auditory syllables. Thus, here we tested whether the representational precision benefit from auditory retrospective attention generalizes across different cognitive demands required at task by further increasing the degree of memory load.

Recent work on visual retrospective attention suggests that the ways in which valid retro-cues benefit recall performance depend on working memory load (Astle et al., 2012). For instance, under low memory load participants re-orient their attention to cued items while maintaining uncued items in memory. However, when memory load is beyond the capacity limit, uncued items seem to be removed from memory without affecting representational precision of the items. Likewise, the precision of visual information retained in working memory can be flexibly controlled only when memory load is within capacity (i.e., low load; Machizawa et al., 2012).

Here, we examine the impact of retro-cues as cognitive resources are constrained by increasingly high memory load. Specifically, we test whether excessive cognitive load beyond capacity may annul any benefits from retro-cues because utilization of retro-cues may require cognitive resources to first reorient attention to, and enhance representational precision of, the cued items. Importantly, given that memory and attentional control capabilities vary widely across individuals (Awh et al., 2007; Rouder et al., 2008), an individual’s precision benefit under varying memory load may depend on her overall memory performance.

## Materials and Methods

### Participants

Thirty-two normal hearing volunteers (18 females, mean age = 24.8 years, age range = 20–30 years) participated. All participants gave written informed consent and were financially compensated. Experimental procedures were in accordance with the Declaration of Helsinki and approved by the local ethics committee of the University of Leipzig.

### Stimuli

Six distinct syllable categories were used (**Table 1**). For each syllable category, there were four naturally varying syllable tokens, recorded by a German female speaker. All tokens were 200 ms in duration and digitized at 44.1 kHz.

**Table 1:** F0 and relative amplification values of the syllable stimuli as resulting from the subjective loudness rating procedure.

Syllable category	Probed syllable F0 (Hz)			Unprobed syllable F0 (Hz)			Relative amplification (dBFS)
	Mean	Minimum	Maximum	Mean	Minimum	Maximum	
/da/	162.6	148.2	177.8	162.1	148.8	176.2	3.26
/do/	180.3	162.9	198.9	183.2	168.3	198.7	7.69
/ge/	174.3	156.8	192.9	174.1	157.8	191.4	4.41
/gu/	188.8	171.5	206.8	190.0	176.7	204.9	7.82
/ko/	184.0	167.3	201.8	186.0	173.5	201.5	6.22
/ti/	192.6	176.6	210.6	190.2	174.1	203.5	0

Two tokens per syllable category served as to-be-probed syllables. For each token, its pitch (F0) was varied in eight steps (±0.125, ±0.375, ±0.75, and ±1.25 semitones) from its original F0 using Praat. The F0-manipulated syllables were presented as probes, and the corresponding original utterances were presented during syllable encoding. In addition, we created an additional set of unprobed syllable sounds using different tokens and pitch variations. These sounds were only presented during encoding to create acoustic variability beyond a fixed stimulus set. For each category, we manipulated the F0s of the two remaining utterances that did not serve as probes; the F0 was manipulated in 10 steps (±0.25 to ±1.25 semitones, in steps of 0.25 semitones). In addition, the F0s of the two syllable utterances used to create probe stimuli were manipulated into four varying steps (±0.5 and ±0.625 semitones). **Table 1** lists the F0 ranges of each syllable category used in the experiment. The F0 dimension of the stimuli was manipulated with Praat version 5.3.

All stimuli were normalized to equivalent amplitude [root-mean-squared dB full scale (RMS dBFS)]. However, it is of note that even with the same physical amplitude across the six syllables, the perceptual loudness may differ due to the manner of articulation and voicing. To match perceptual loudness of the six syllables, RMS normalization incorporated relative intensity amplification based on the subjective loudness rating data of the syllables collected from an independent set of participants (*n* = 8; see **Table 1**). On each trial of this syllable loudness rating procedure, a syllable stimulus was presented under a speech-shaped noise masker played in the background (see below). Amplitude of the background noise was fixed at 50 dB full scale (dBFS), and the relative amplitude of the syllables varied from ± 9 dB (in 3 dB steps). On each trial, participants provided subjective loudness rating of the syllable, scaled from 1 (very quiet) to 9 (very loud). Average dBFS of each syllable category that was rated as average loudness (i.e., rating score 5) was used for adjustment of relative amplification.

To equate the number of auditory items in all trials, presentations of syllables in 2- and 4-syllable sequences were flanked by noise bursts. The noise-burst filler emulated the syllables’ temporal characteristics and was created by applying the average temporal amplitude envelope of all syllable stimuli used in the task to low-pass filtered (cut-off 8 kHz) white noise. As the syllable stimuli, the noise-burst filler was 200 ms in duration and had a sampling rate of 44.1 kHz. Finally, amplitude of the noise-burst filler was matched to that of /ti/ syllable (i.e., no intensity amplification) presented during encoding.

Furthermore, a continuous speech-shaped noise masker (10.4 s duration) was played in the background. The masker was created by filtering a broadband white noise to approximate the long-term average spectrum of speech (Wilsch et al., 2015). The filter was created based on frequency spectrum of speech sounds (i.e., 60 concatenated German nouns spoken by a female speaker).

### Experimental Task

**FIGURE 1:**
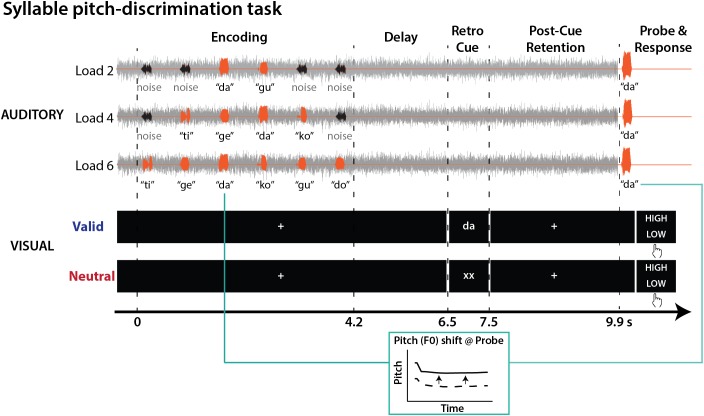
Syllable-pitch discrimination task structure. On each trial, speech syllables (orange) and noise bursts (black) were presented against a speech-shaped noise masker (gray). Participants judged whether the pitch of the probe syllable was higher or lower in comparison to the same category syllable heard during encoding. During memory retention, either a valid (e.g., “da”) or neutral (“xx”) visual retro-cue was presented.

**Figure 1** illustrates the syllable pitch-discrimination task implemented within a retro-cueing paradigm. We used a 2 × 3 design to manipulate factors of visual *retro-cues* (valid vs. neutral) and *memory load* (2, 4, or 6 syllables to-be-retained).

On each trial, participants encoded a sequence of two, four, or six distinct auditory syllables with a 0.6-s inter-stimulus interval, while seeing a fixation cross. After a 2.3-s delay period, a 1-s visual retro-cue was presented. The cue was followed by a post-cue retention phase (2.4 s) before the presentation of an auditory probe. One of the syllables heard during encoding was presented as a probe, but with a slight change in pitch (F0). Participants judged whether the probe syllable pitch was higher or lower in comparison to the pitch of the corresponding syllable presented during encoding.

Two types of retro-cue were presented. Firstly, a valid retro-cue provided information about category identity of the probe. A written syllable (e.g., “da”) was presented to direct participants’ attention to one of the syllables maintained in memory. Secondly, a neutral retro-cue (i.e., “xx”) did not provide any information about the probe.

Participants performed the task under a speech-shaped noise masker. To reduce any confounding factors due to the difficulties in encoding the probe stimulus, the probe syllable was not masked by the noise. The noise masker and probe stimuli were delivered at 50 dB above the individual’s sensation level (SL). Intensity of the syllable sequence [i.e., signal-to-noise ratio (SNR)] was individually adjusted using an adaptive tracking procedure (Levitt, 1971; see below).

To minimize the sequential position effect (i.e., primacy/recency effects), we only analyzed the trials that probed the syllable in the 3^rd^ or 4^th^ position in the sequence presented during encoding (75% of trials). In the remaining catch trials, we probed syllables in other positions (for 4 and 6 load conditions); these were later excluded from data analysis, but presented only to prevent participants from directing their attention to specific sequence positions during encoding. Post-experiment questionnaire responses confirmed that participants were not aware of this manipulation.

Participants went through 12 blocks (× 32 trials). Within each block, all conditions (i.e., 2 retro-cues × 3 memory loads) were randomized (four trials each) with an additional eight catch trials. The experiment was controlled by Presentation software (Neurobehavioral Systems).

### Individual Adjustment of Acoustic Stimuli

In auditory research, it is good practice to control differences in stimulus audibility across listeners in speech-in-noise tasks (Wöstmann et al., 2017). To this end, we controlled differences in audibility of syllable sequence embedded in background noise. We estimated the SNR that yielded an equivalent level of performance in the syllable pitch-discrimination task in each individual. In detail, prior to the main experiment, participants performed the presumably most challenging condition of the syllable pitch-discrimination task (i.e., detecting a pitch change in one of the six syllables presented during encoding without any retro-cue). Using an adaptive tracking procedure (one-up one-down to track approximately 50% performance; Levitt, 1971), we adjusted the intensity of the to-be-encoded syllable sequence. The intensities of the background noise masker and the probe were fixed at 50 dB SL, and the intensity of the syllable sequences were adjusted starting from 0 dB SNR. The adjustment step size was initially set at 5 dB, and reduced to a minimum of 0.5 dB toward the end of the block. Participants completed three blocks (30 trials per block) of the adaptive tracking procedure. The individual threshold was determined by taking average intensity across the three tracking blocks (for a similar application of adaptive tracking in a speech-in-noise task, see Wöstmann et al., 2015). The resulting average SNR across participants was -13.8 dB (*SD* = 3.1).

### Working Memory Span

We assessed participants’ working memory span using the well-established backward auditory digit span test (Wechsler, 1997). On each trial, participants heard a sequence of spoken digits (1–9), and participants verbally repeated the digit sequence in a reverse order. The test consisted of seven levels of sequence length, which increased from 2- to 8-digit sequences. Two items (i.e., sequence lists) on each level were tested. An item was counted as correct only if all digits in the item were recalled in the correct order. The test ended when an individual incorrectly recalled both items at a given level. The digit span score possibly ranges from 0 to 14.

### Procedure

The experiment was divided into two separate sessions. On the first session, participants first practiced the task (18 trials) without any background noise. The practice session was repeated if performance was <70%. Next, we adjusted individuals’ SNRs, which was followed by the first half of the syllable-pitch discrimination task. On the second session, participants completed the second half of the syllable-pitch discrimination task. Afterward, we assessed participants’ working memory span via the backward auditory digit span test and a short post-test questionnaire.

### Data Analysis

In all measures, we first assessed the overall effect of the retro-cue (i.e., valid vs. neutral trials) collapsed across the three memory load conditions. This approach was taken to examine whether the retro-cue benefit generalizes across differing memory loads based on precisely estimated performance measures of the retro-cue conditions with sufficient amount of trials (Macmillan and Creelman, 2004). Next, we evaluated the effects of the retro-cue in respect to the memory load conditions (i.e., retro-cue × memory load interaction).

Any significant effects found in the repeated-measures ANOVA were followed by pairwise *post hoc t*-tests.

#### Behavioral Measures

Participants’ response time (log-transformed) of correct trials and bias-free sensitivity *d′* (Macmillan and Creelman, 2004) were analyzed separately.

#### Psychophysical Modeling

We further assessed a fine-grained measure of perceptual precision using a psychophysical modeling approach (Bays and Husain, 2008; Zhang and Luck, 2008; Murray et al., 2013; Lim et al., 2015). We fitted each individual’s response patterns to F0 changes of the probe with a psychometric (sigmoid) function:

$$\begin{array}{c} y=\frac{1}{1+e^{-k(x-m)}} \end{array}$$

where *y* indicates the proportion of “high” responses to the probe and *x* indicates the F0 changes of the probe. Parameters of slope (*k*) and inflection point (*m*) of the curve were estimated based on a non-linear least-square fitting procedure (*lsqcurvefit* in MATLAB). The inflection point *m* estimates participants’ response bias. The slope parameter *k* reflects the perceptual precision in a participant’s responses; the steeper the slope, the higher the precision in judging the pitch of the probe (**Figure 4A**).

As done for the other behavioral measures, we first assessed the overall effect of the retro-cue. Then, we evaluated the effects of the retro-cue in respect to memory loads. Accordingly, we first estimated slope *k* and bias *m* parameters from response patterns of the valid and neutral retro-cueing conditions (collapsed across memory load conditions). Next, to examine the effect of retro-cues as a function of memory loads, we estimated *k* and *m* for each of the retro-cue × memory load conditions^1^. Prior to all statistical analyses, the slope (*k*) estimate was log-transformed (ln *k*) to ensure normality.

#### Changes in Retro-Cue Benefits across Memory Loads

Depending on the amount of auditory syllables held in memory, the benefit from valid (vs. neutral) retro-cues may differ. Thus, we assessed how an individual’s perceptual precision benefit from valid retro-cues changes across varying memory loads. For each load, perceptual precision benefit from valid retro-cues was expressed as difference in log-transformed slope estimates (ln *k*_V alid_–ln *k*_Neutral_). For each participant, we quantified a linear-trend coefficient that characterizes changes in cue-related precision benefits as a function of memory load (using *polyfit* in MATLAB).

Using a one-sample *t*-test against zero, we tested whether precision benefits from valid retro-cues change (i.e., linear-trend coefficient) across memory loads. We further examined the correlation of individual differences in the change of cue-related precision benefit and overall memory performance (i.e., sensitivity *d′*). To ensure the robustness of the correlation, 95% confidence intervals (CIs) were generated from 1000 iterations of bootstrapped correlations.

## Results

### Performance Benefits from Valid Retro-Cues

**FIGURE 2:**
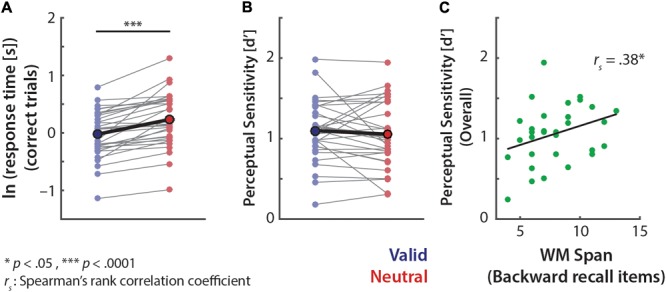
Overall pitch-discrimination performance. Log-transformed response time of correct trials **(A)** and perceptual sensitivity *d′* **(B)**. Data points connected by thin lines represent individuals’ performances. Mean performances are indicated by bold circles and bold lines. **(C)** Correlation between individuals’ backward auditory digit span scores and overall memory performances (i.e., overall *d′* collapsed across all retro-cue conditions).

A *t*-test on the log-transformed response time on correct trials revealed that participants responded significantly faster in the valid than neutral retro-cue trials (*M*_valid-neutral_ = -20.8% in response time (s), *t*_31_= 6.28, *p* < 0.0005, *r* = 0.75; **Figure 2A**). However, there was no significant effect of retro-cue on overall sensitivity *d′* (*t*_31_= 0.88, *p* = 0.39; *r* = 0.16; **Figure 2B**).

We further examined whether individuals’ working memory span was related to overall memory performance, and to the extent of recall benefits from the valid (vs. neutral) retro-cues. To this end we correlated individuals’ scores on the well-established backward auditory digit span test (Wechsler, 1997) and their memory performance (*d′*). As shown in **Figure 2C**, the relationship was significantly positive (*r*_s_= 0.38, *p* = 0.033), which indicates that individuals with higher memory spans exhibited better syllable-pitch memory performance. However, overall performance benefits from valid retro-cues (i.e., *d′*_V alid_–*d′*_Neutral_) did not show any relationship to the backward digit span score (*r*_s_= 0.03, *p* = 0.88).

**FIGURE 3:**
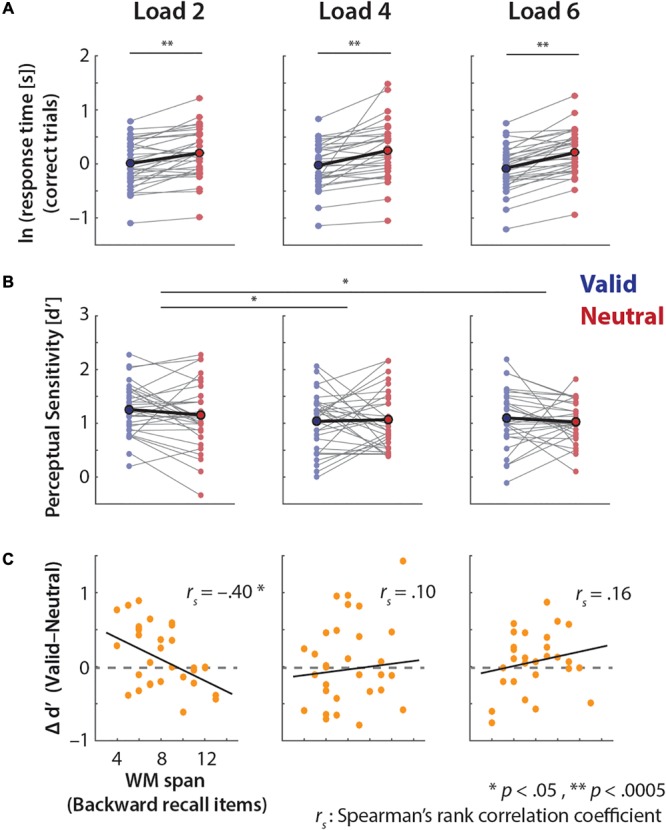
Pitch-discrimination performances of the valid and neutral retro-cue trials in each memory load. **(A)** Log-transformed response time exhibits a consistent effect of retro-cue across memory loads. **(B)** Perceptual sensitivity *d′* shows higher overall performance in the low load (Load 2) than higher loads (Loads 4 and 6). The illustration scheme is same as **Figure 2**. **(C)** Correlations between individuals’ backward auditory digit span scores and the cue-related *d′* differences (*d′*_V alid_–*d′*_Neutral_) in each memory load condition.

Next, we evaluated whether the effect of retro-cue on these behavioral measures varied across memory loads (**Figure 3**). A 2 × 3 ANOVA on the log-transformed response time revealed a main effect of retro-cue (*F*_1,31_= 44.97, *p* < 0.0005), but no significant main or interaction effects related to memory load (memory load: *F*_1.94,60.13_= 1.45, *p* = 0.24; retro-cue × memory load: *F*_1.85,57.4_= 1.90, *p* = 0.16).

In contrast, the 2 × 3 ANOVA on perceptual sensitivity *d′* revealed a significant main effect of memory load (*F*_2,62_= 3.58, *p* = 0.034, $\eta_{p}^{2}$ = 0.10), but no significant effects related to retro-cue (retro-cue: *F*_1,31_= 0.78, *p* = 0.38, $\eta_{p}^{2}$ = 0.03; retro-cue × memory load: *F*_1,62_= 0.45, *p* = 0.64, $\eta_{p}^{2}$ = 0.01). *Post hoc t*-tests on overall memory performances (*d′*) across memory loads revealed that participants performed significantly better in Load 2 than Load 4 (*t*_31_ = 2.50, *p* = 0.018, *r* = 0.42) and Load 6 (*t*_31_ = 2.60, *p* = 0.014, *r* = 0.43); Load 4 and Load 6 performances were comparable (*t*_31_= 0.23, *p* = 0.82, *r* = 0.042).

As noted, we did not find a relationship between individuals’ backward digit span scores and the degree of overall memory performance benefits from the valid retro-cues (i.e., *d′*_V alid_–*d′*_Neutral_). However, when we further examined this relationship at each memory load, only for Load 2 backward digit span score exhibited a significant negative relationship to performance benefits from valid retro-cues (**Figure 3C**; *r*_s_= -0.40, *p* = 0.024). This indicates that, at this lower load, individuals with lower working memory spans benefit more from a valid retro-cue. In keeping with this observation, the relationship trended in the opposite, positive direction for the higher-load conditions (**Figure 3C**).

### Retrospective Attention Enhances Perceptual Precision

**FIGURE 4:**
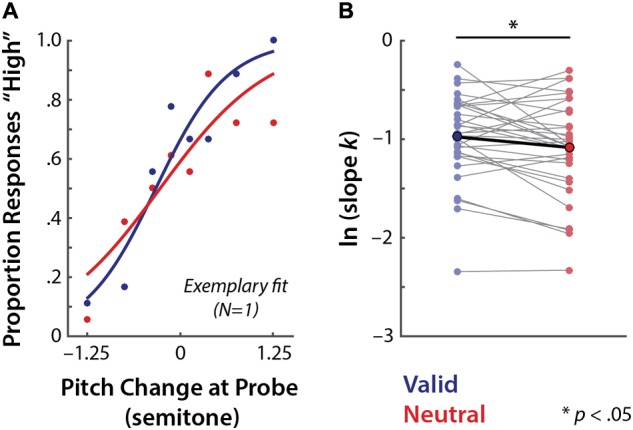
Perceptual precision in valid vs. neutral retro-cue conditions. **(A)** An exemplary (*N* = 1) psychophysical modeling results. Dots represent the actual data points and lines indicate model-estimated fits (see Supplementary Figure S1 for all individual participants’ fits). **(B)** All (*N* = 32) individuals’ overall perceptual precision estimates of the valid and neutral retro-cue trials (thin gray lines; ln *k*) and the mean across individuals (bold line).

Using psychophysical modeling we estimated each individual’s response bias (inflection/mid-point parameter, *m*) and perceptual precision (slope parameter, *k*) in the pitch-discrimination task (**Figure 4A**).

The overall response bias estimate (*m*) did not differ significantly between the valid and neutral retro-cue trials (*t_31_*= 0.87, *p* = 0.39), and neither condition exhibited any significant bias (one-sample signed-rank tests against 0; all *t*s ≤ 1.87, *p*s ≥ 0.07). Further examinations of the model-estimated bias including the memory load condition did not reveal any significant effects of retro-cue or memory load (all *F*s ≤ 1.00, *p*s ≥ 0.33). Also, none of the 2 (retro-cue) × 3 (memory load) conditions exhibited significant bias (one-sample tests against 0; all *ts* < 0.96; *p*s > 0.35).

Based on our previous study (Lim et al., 2015), we strongly expected higher precision in the valid than the neutral retro-cue trials. Consistent with this expectation, planned comparison revealed that the log-transformed slope parameter *k* (ln *k*) showed a significant difference between the retro-cue conditions: overall perceptual precision was indeed significantly higher for the valid than neutral retro-cue condition (*t_31_* = 2.12, one-tailed *p* = 0.021, *r* = 0.36; **Figure 4B**).

Given the significant precision benefits from valid retro-cues, we further examined whether the degree of the benefit changes across memory loads. A 2 × 3 ANOVA on perceptual precision (ln slope *k*) revealed a marginal effect of retro-cue (*F*_1,31_ = 3.08, *p* = 0.09), but no significant main or interaction effect related to memory load (memory load: *F*_1.62,50.31_ = 1.02, *p* = 0.35; retro-cue × memory load: *F*_1.85,57.4_ = 0.45, *p* = 0.63). Thus, perceptual precision did not differ across memory loads.

**FIGURE 5:**
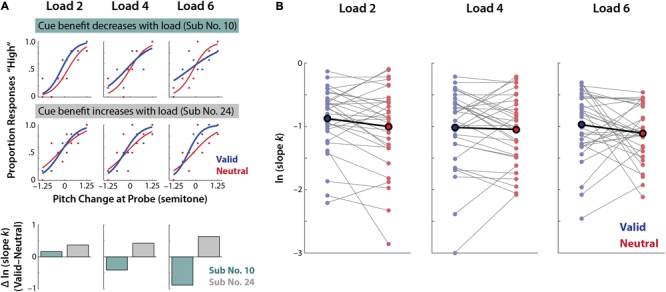
Inter-individual differences in perceptual precision benefits from valid vs. neutral retro-cues in each memory load condition. **(A)** Psychophysical modeling results of two individuals exhibiting different trends of precision benefits from valid retro-cues. In each memory load condition, psychophysical model parameters are separately estimated for the valid and neutral retro-cue conditions. (Top) An individual exhibiting a decrease in cue-related precision benefit as memory load increases (i.e., blue slope becomes shallower with higher memory load). (Middle) An individual exhibiting the reverse pattern; an increase in cue benefits as memory load increases. (Bottom) The change of cue-related precision benefits (ln *k*_V alid_–ln *k*_Neutral_) of the two individuals exhibiting an opposite pattern across loads. **(B)** Individual subjects’ perceptual precision (ln *k*) in the valid and neutral retro-cue conditions per memory load (thin gray lines) and the mean across individuals (bold black line).

As **Figure 5** illustrates, this lack of consistent group-level effects may be due to high inter-individual variability in the extent of benefits from valid retro-cues across loads.

Although ANOVA is robust against the violation of normality assumptions, we have found a modest non-normality in the dependent variable of model-fitted parameters (using Shapiro–Wilk test *p* < 0.05). To account for this, we repeated our analyses using non-parametric tests; we found highly consistent results with the ones found using parametric tests. Overall perceptual precision was significantly higher for the valid than neutral retro-cue condition (*Z* = 2.08, one-tailed *p* = 0.019, *r* = 0.26). When we further examined whether the degree of the benefit changes across memory loads, a Friedman test revealed no significant main or interaction effects of either condition (all χ^2^s ≤ 2.31; *p*s ≥ 0.29)^2^.

### Individual Benefit from Valid Retro-Cues Depends on Memory Performance

**FIGURE 6:**
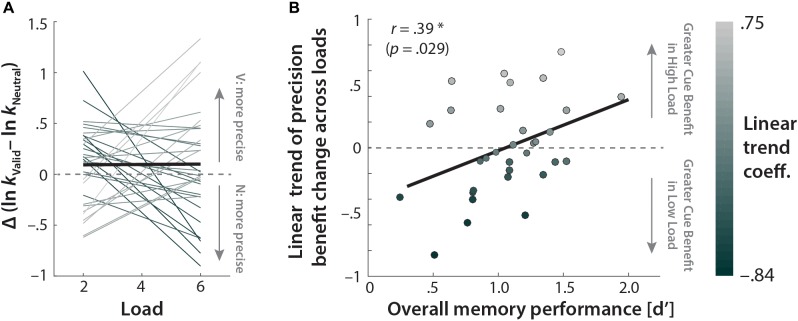
Memory load affects individual differences in the amount of cue-related precision benefit. **(A)** Thin colored lines indicate individual participants’ linear trends for the effect of memory load (2, 4, 6 syllables in memory) on the cue-related precision benefit (ln *k*_V alid_–ln *k*_Neutral_). Line colors correspond to individuals’ linear-trend coefficients. The bold black line indicates the average linear trend. **(B)** Correlation between individual’s overall memory performance (*d′*) and the linear change in the cue benefits with increased memory load. The colors of the dots correspond to individuals’ (*N* = 32) linear-trend coefficients as in **(A)**.

Given the high inter-individual variability in valid cue-related precision benefits (**Figure 5**), we examined how individuals’ precision benefit changes as a function of memory load. We estimated individuals’ linear-trend coefficient for the change in the amount of precision benefits (i.e., ln *k*_V alid_–ln *k*_Neutral_) with increasing memory load. A one-sample *t*-test against 0 on these individual trend coefficients revealed that cue-related precision benefit did not change across loads (*t*_31_ = 0.054, *p* = 0.96, *r* = 0.010). The lack of precision change at the group level was due to high individual differences in the amount and sign of cue-related precision benefit change as a function of memory load (**Figure 6A**).

We further examined whether this high individual variance depended on individuals’ overall memory performance (*d′*). The correlation analysis between the susceptibility of the cue benefit to load on the one hand and participants’ overall working memory performance (*d′*) on the other hand revealed a significant positive relationship (*r* = 0.39, *p* = 0.029; **Figure 6B**; bootstrapped 95% CIs = [0.02, 0.65]); this indicates that as memory load increased, individuals with high working memory performance benefitted more from valid retro-cues. In contrast, low-performing individuals showed the strongest cue benefit when memory load was relatively low.

## Discussion

Here we investigated, first, whether the precision benefit from retrospective attention changes as a function of memory load. Second, we explored whether this change of retrospective attention benefit across varying loads depends on an individual’s working memory performance. To do so, we utilized an auditory retro-cueing task where participants retained a varying number of auditory syllables in memory.

### Valid Retro-Cues Facilitate Memory Performance and Recall Precision

We found that valid retro-cues were generally advantageous (i.e., across varying memory loads) for pitch discrimination of the cued item. In line with previous findings (Griffin and Nobre, 2003; Kumar et al., 2013; Souza et al., 2014; Backer et al., 2015), our participants were significantly faster in making accurate syllable pitch-change judgments (i.e., response time) when their attention was re-directed to the to-be-probed syllable item in memory. Thus, participants do utilize the information provided by valid retro-cues.

Here, we aimed to replicate and extend this core, previous finding (Lim et al., 2015) under varied memory load. It is of note that participants’ overall memory performance (i.e., perceptual sensitivity *d′*) did not exhibit a significant benefit from valid retro-cues. This performance measure results from aggregating across the varying amounts of pitch change occurring at the probe syllable. Thus, the lack of significant effect of retro-cue might show that overall *d′* is not a sensitive enough measure to capture the benefit that valid cues can yield (e.g., Griffin and Nobre, 2003; Lepsien and Nobre, 2007; Astle et al., 2012 on showing a stronger effect of retro-cue on response time than accuracy measure).

However, by using a psychophysical modeling approach (Bays and Husain, 2008; Zhang and Luck, 2008; Murray et al., 2013; Lim et al., 2015), we observed that valid (vs. neutral) retro-cues were generally beneficial in enhancing representational precision of auditory syllable recall. Thus, compared to overall memory performance (*d′*), the psychophysical modeling approach provides a more fine-grained measure of precision of syllable pitch judgment across different amounts of pitch change. Concomitantly, neural oscillatory modulation in Lim et al. (2015) only correlated with the modulation in perceptual precision (ln slope *k*) but not in *d′*.

However, it is of note that this representational precision enhancement for the cued item was small. As memory load increased, precision benefits were only observed in participants with higher memory capacity (**Figure 6B**). This suggests that a benefit from retro-cues may itself require cognitive resources. This interpretation is consistent with our previous electrophysiological study, in which we found that selective attention to auditory items in memory engages neural resources to actively maintain a precise representation of the attended item (Lim et al., 2015). Thus, a retro-cue is not effective in and of itself; but it is only effective if the neural system of the observer actively uses the cue to re-orient attention. This is in accord with the view that reorienting attention to items in memory may recruit additional neural processes (Griffin and Nobre, 2003). In sum, utilization of retro-cues may require extra cognitive resources to process the cue and to reorient attention, which in turn, can enhance representational precision of the cued items.

### Individual Differences in Valid Retro-Cue Usage

Participants exhibited high variability in the extent to which they benefitted from valid retro-cues across varying memory load. On average, precision benefit from valid retro-cues was not evident in the higher memory load conditions; however, some participants benefitted more (in terms of perceptual precision) from valid retro-cues as memory load increased, whereas others benefitted only at lower memory load.

Which factors could explain such differences? The precision with which internal representations are held in memory depends on available cognitive resource (e.g., Wilken and Ma, 2004; Machizawa et al., 2012; for reviews see Ma et al., 2014; Bays, 2015). Also, the use of retro-cues requires cognitive resources. If so, we expect that varying levels of working memory capacity and capabilities across individuals should play roles in determining the degree to which valid retro-cues benefit representational precision of the attended items in memory. Indeed, individual working memory performance (*d′*) was related to how cue-related precision benefit changed with increasing memory load (**Figure 6B**). We observed that individuals with high memory performance exhibited greater precision benefit from valid retro-cues as memory load increased. In contrast, relatively low performing individuals exhibited increased cue-related benefit with lower memory load.

Based on our results, the following mechanism emerges: high-performing individuals have enough capacity (i.e., cognitive resources) to maintain a relatively low number of to-be-remembered items in memory well. Thus, valid retro-cues are not additionally facilitatory under low memory load. They become efficient (i.e., they save more resources than their incorporation would cost) only when memory load limits cognitive resource. Conversely, low-capacity individuals benefit from retro-cue information already at lower degrees of memory loads. But once their own memory capacity is exceeded by the amount of to-be-remembered items, they cannot flexibly reallocate cognitive resources further (see Unsworth and Robison, 2015 for pupillometry evidence).

Supporting this interpretation, we have observed that the backward digit span score predicted the pitch-discrimination accuracy from valid retro-cues in low memory load (**Figure 3C**); the lower the memory span, the greater the retro-cue benefit. At higher memory loads, however, this effect was absent but toward an opposite direction—that is, greater retro-cue benefit for higher span individuals.

Moreover, the relatively low working memory performance across participants (mean *d′* across all conditions: M ± SD = 1.07 ± 0.36; cf. Cowan, 2001) is of note. Presumably, the high task complexity and its demands may have resulted in overall low memory performance. The current study required participants to encode and maintain auditory syllables in the midst of constant background noise for a relatively long time period (∼10 s). The presence of background noise and prolonged memory retention are known to impact working memory representations and recall performance (Rabbitt, 1968; Wilsch and Obleser, 2016). In addition, good (e.g., either semantic or temporal) predictions about the task facilitates encoding and retention of sensory information, thereby reducing cognitive load/demands (Rohenkohl et al., 2012; Wilsch et al., 2015; Wöstmann et al., 2015). However, participants in the current study could not form any prediction about upcoming trial structure because manipulations of memory load and validity of the retro-cue were completely randomized.

We presume that all of these additive task demands may have pushed participants toward their limit (i.e., own memory capacity), but with different amounts of resulting or *effective* memory load across individuals. Thus, since the use of valid retro-cues requires a certain expense of cognitive resources, our results suggest that participants make use of the cue in a well-adapted fashion when necessary.

### Underlying Neural Mechanisms of Retrospective Attention

The observed individual differences in the usage of valid retro-cues (**Figures 5, 6**) could reflect the interplay between the use of exogenous retro-cues and allocation of endogenous neural resources. At the neural level, the amplitude of neural alpha oscillations (∼7–13 Hz) in the magneto-/electroencephalogram (M/EEG) is suggested to be a sensitive marker of cognitive resources allocated to a task: alpha power increases with higher memory load (Jensen et al., 2002; Tuladhar et al., 2007; Obleser et al., 2012). During selective attention, alpha power increases and decreases in brain areas processing task-irrelevant and relevant information, respectively (Strauß et al., 2014; de Pesters et al., 2016; Wöstmann et al., 2016).

Our previous studies also observed that the magnitude of neural alpha enhancement predicts an individual’s precision benefit from valid cues (Lim et al., 2015; Wilsch et al., 2015). These findings indicate that allocation of neural resources (i.e., alpha power enhancement) in non-informative neutral cues trials can achieve working memory performance comparable to that achieved by individuals using exogenous valid retro-cues. Therefore, individuals may adopt different cue usage to optimally balance neural resources required for exogenous vs. endogenous processes.

## Conclusion

Using psychophysical modeling, we have demonstrated that selective attention to auditory working memory facilitates recall and enhances perceptual precision of the attended item in a cognitively challenging task. Importantly, the extent to which an individual benefits from retrospective attention across varying memory loads is linked to her overall working memory performance. Thus, our findings suggest that the processing of informative retro-cues in order to re-orient attention itself necessitates the allocation of cognitive resources.

## Author Contributions

S-JL and JO designed the experiment; S-JL and FG prepared the materials and performed the experiment; S-JL, MW, and JO analyzed the data and wrote the manuscript. All authors discussed the results and edited the manuscript.

## Conflict of Interest Statement

The authors declare that the research was conducted in the absence of any commercial or financial relationships that could be construed as a potential conflict of interest.

## Footnotes

1 To avoid overfitting of the data while at the same time avoiding condition-specific biases, we restricted the bounds of k and m for each memory load × retro-cue condition to the range of individual parameter estimates obtained for k and m when fitting the overall response pattern collapsed across all conditions (i.e., a subject-specific grand average).

2 In order to accommodate the limitation of the Friedman test on examining an interaction effect, we tested the effect of memory load on the difference measure (i.e., Valid–Neutral retro-cue) of each memory load.
