# Supplementary material for: The Benefit of Attention-to-Memory Depends on the Interplay of Memory Capacity and Memory Load
Source: Front Psychol. 2018 Feb 19;9:184. doi: 10.3389/fpsyg.2018.00184 (PMC5827549; doi:10.3389/fpsyg.2018.00184)
Supplement: Supplementary file 1 [file Image_1.PDF]

Supplementary Material

The benefit of attention-to-memory depends on the interplay of memory capacity and memory load

Sung-Joo Lim *, Malte Wöstmann, Frederik Geweke, Jonas Obleser*

*** Correspondence:**

Sung-Joo Lim & Jonas Obleser

(sungjoo@bu.edu; jonas.obleser@uni-luebeck.de)

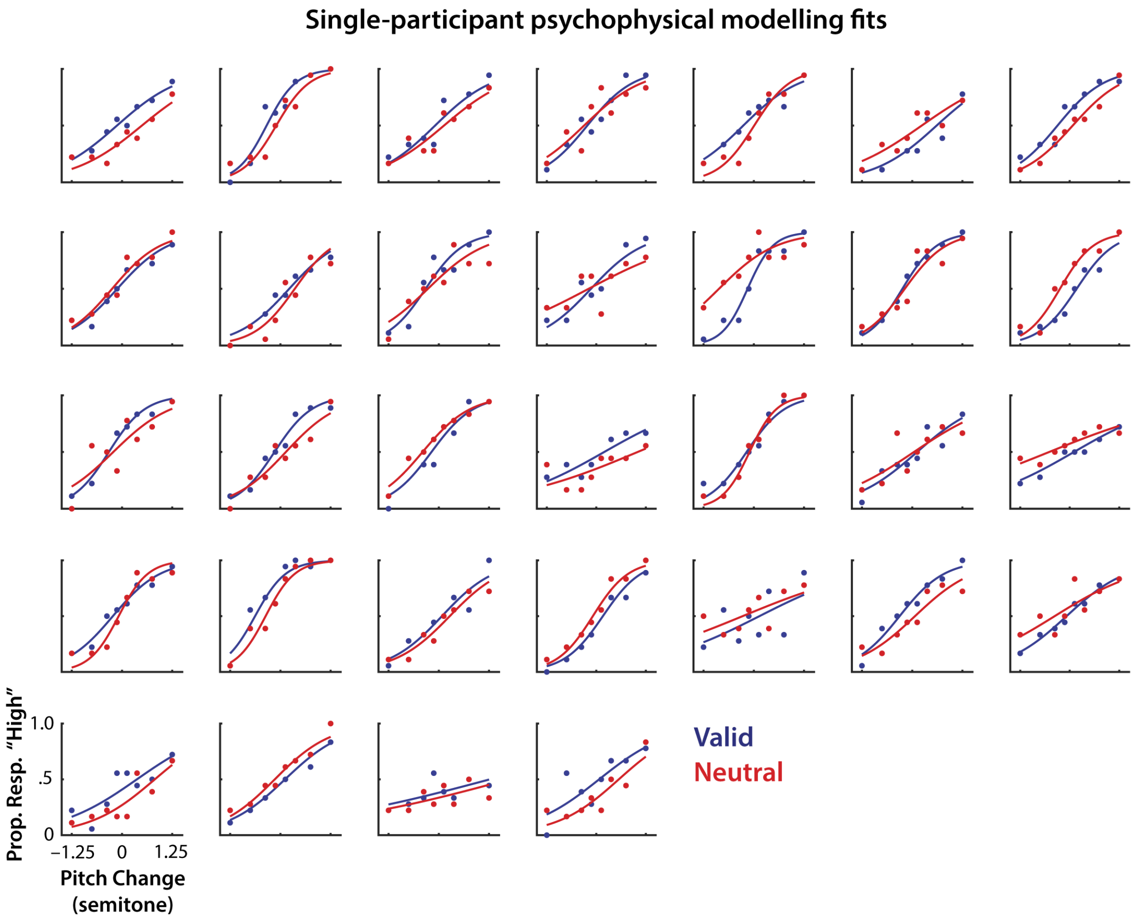

Figure S1. Single-participant model-estimated fits. Dots represent the proportion of "high" responses (y-axis) as a function of pitch change at probe (x-axis). The lines indicate model-estimated fits.
